# Malaria epidemiology and anti-malarial drug efficacy in Guinea: a review of clinical and molecular studies

**DOI:** 10.1186/s12936-021-03809-9

**Published:** 2021-06-16

**Authors:** Mahamoud Sama Cherif, Prabin Dahal, Abdoul Habib Beavogui, Alexandre Delamou, Eugene Kaman Lama, Alioune Camara, Mamadou Pathe Diallo

**Affiliations:** 1 Faculty of Sciences and Health Technics, Gamal Abdel Nasser University of Conakry, Conakry, Guinea; 2 Centre National de Formation et Recherche en Sante Rurale de Maferinyah, Maferinyah, Guinea; 3 Centre for Tropical Medicine and Global Health, Nuffield Department of Medicine, University of Oxford, Oxford, UK; 4 National Malaria Control Programme (NMCP), Conakry, Guinea

**Keywords:** Malaria, Guinea, Resistance, Artemisinin-based combination therapy, Efficacy, *Plasmodium falciparum*

## Abstract

Malaria is one of the leading causes of mortality and morbidity in Guinea. The entire country is considered at risk of the disease. Transmission occurs all year round with peaks occurring from July through October with *Plasmodium falciparum* as the primary parasite species. Chloroquine (CQ) was the first-line drug against uncomplicated *P*. *falciparum* in Guinea until 2005, prior to the adoption of artemisinin-based combination therapy (ACT). In this review, data on therapeutic efficacy of CQ and artemisinin-based combinations reported in published literature is summarized. Against CQ, a failure rate of 27% (12/44) was reported in a study in 1992; a median failure rate of 15.6% [range: 7.7–28.3; 8 studies] was observed during 1996–2001, and 81% (17/21) of the patients failed to clear parasitaemia in a study conducted in 2007. For artemisinin-based combinations, three published studies were identified (1495 patients; 2004–2016); all three studies demonstrated day 28 polymerase chain reaction corrected efficacy > 95%. One study characterized *kelch*-*13* mutations (389 tested; samples collected in 2016) with no evidence of mutations currently known to be associated with artemisinin resistance. The impact of the ongoing COVID-19 pandemic and widespread usage of counterfeit medicines are immediate challenges to malaria control activities in Guinea.

## Background

**Fig. 1:**
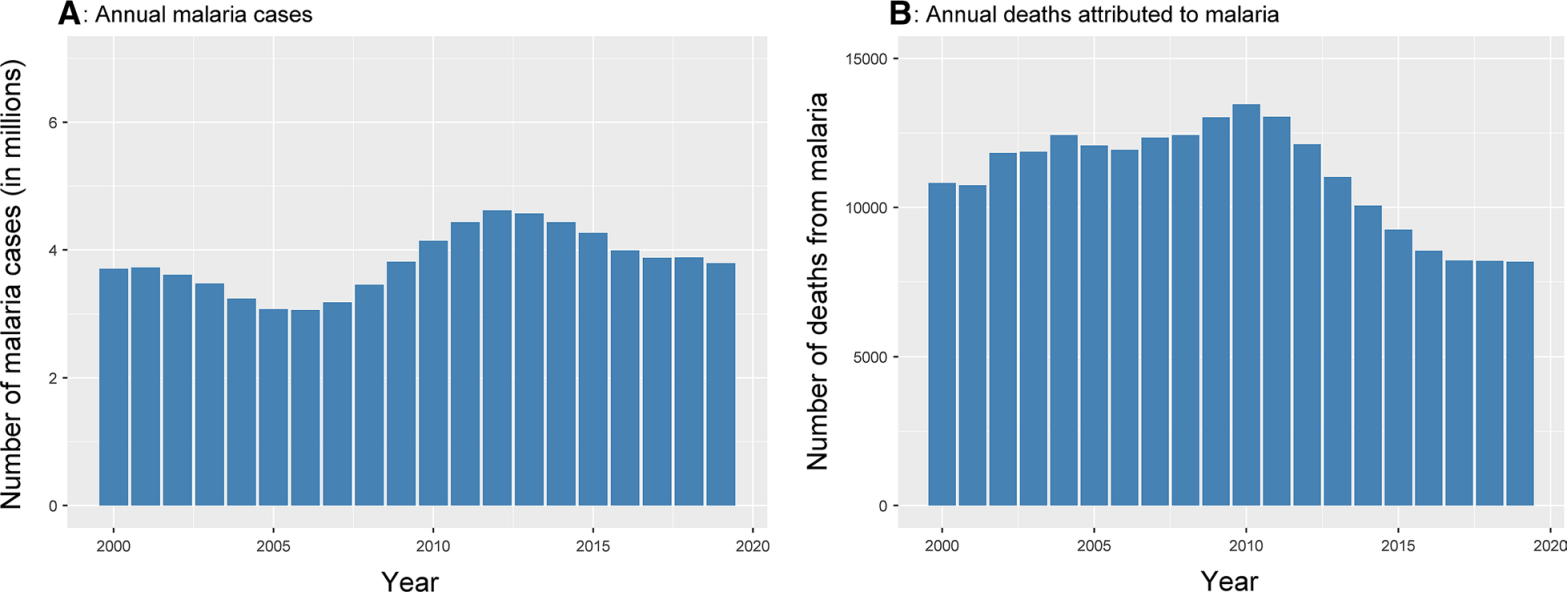
Total malaria cases and deaths in Guinea. Data are from WHO World Malaria Report 2020 [3]

Malaria is one of the leading causes of mortality and morbidity in Guinea with the entire population at risk of the disease [1]. In 2016, malaria was responsible for 31% of all out-patient consultations [2]. Malaria control has remained one of the top-most public-health priorities. There has also been a recent expansion in international support towards malaria control; Guinea received a funding of 30 million USD in 2017 and a further 45 million USD in 2019 [3]. The increased funding has led to a distribution of 2.7 million courses of artemisinin-based combination therapy (ACT) in 2017 and a further 1.8 million courses in 2018 [3]. Just under 3 million rapid diagnostic tests (RDTs) were distributed annually (2017–2019), and long-lasting insecticidal nets (LLINs) and/or indoor residual spraying (IRS) coverage was between 50 and 80% in 2019 [3]. There is currently a surplus of insecticide-treated nets (ITNs), anti-malarial drugs, and RDTs in combatting malaria for the next 2 years [4]. Despite these important achievements, malaria remains a major public health problem. The disease burden is still high with an estimated 3.8 million cases and 8180 malaria-attributed deaths in 2018 (Fig. 1A. B) [3].

This review discusses the overall epidemiological features of malaria in Guinea, presents the evolution of national malaria control programme (NMCP), highlights major milestones achieved, summarizes clinical and molecular data from published literature, and discusses some immediate challenges and future areas of focus.

### Epidemiological features of malaria in Guinea

#### Parasite species

*Plasmodium falciparum* is the primary cause of malaria in Guinea. Non-falciparum malaria is rare with sporadic descriptions in published literature [5, 6]. Cases of *Plasmodium vivax*, *Plasmodium ovale*, and mixed *P. falciparum* and *P. vivax* infections have been reported among travellers [5, 7–9].

#### Transmission

The country is divided into four main ecological regions: Lower Guinea lies in the west along the coast, the Middle Guinea is a mountainous region with cooler temperature, the Upper Guinea lies to the northeast and the Forest Guinea is located in the southeast [10]. There is a regional variation in malaria endemicity with hyper-endemic transmission in the southern forested region [5], and holo-endemic transmission in the lower and eastern region [10]. Malaria transmission occurs all year round with peaks occurring from July through October.

#### Vector distribution

The main vectors responsible for malaria transmission are *Anopheles funestus*, *Anopheles gambiae*, *Anopheles arabiensis* with transmission occurring from dusk to dawn [11]. *Anopheles gambiae* was found to be the main vector in Fouta Djallon (Middle Guinea) in the 1980s [12]. In the Forest Guinea region, *An. gambiae *sensu lato (*s.l*.) is the main vector followed by *An*. *funestus* [10]. In Conakry, *An. gambiae *sensu stricto (*s.s*.) remains the most abundant vector [13].

#### Risk factors

Children under the age of five bear the largest burden of the disease in the country. A 2014 nationwide cross-sectional survey estimated an overall prevalence of 44% (range: 38–61%) among children less than 9 years [5]. Risk factors include: those living in the Forest Guinea region or in rural areas, older children looked after by farmers or housewives, and those with splenomegaly [5]. Among pregnant women, risk factors include: those not using LLINs, those with sub-optimal antenatal care visits and taking incomplete sulfadoxine-pyrimethamine (SP) doses [14]. There is also a high prevalence of malaria among infants (21.7% among infants less than 6 months old [15]).

#### Burden of the disease

An estimated 3.7 million cases occurred in 2000 and the annual case burden has remained approximately constant in the ensuing decades (Fig. 1) [3].

### Chronology of malaria control efforts in Guinea

The Guinean government drafted the first ever policy to combat malaria in 1970 and chloroquine (CQ) was formally adopted as the first line therapy (Table 1). In the 1970s, vector control measures were introduced with an overall aim of eradicating malaria from the country. In the 1980s, Guinea adopted an integrated project to fight against communicable childhood diseases (including malaria). The national guidelines for treating malaria was also updated during this decade and CQ was adopted as a prophylactic treatment in pregnant women. In the 1990s, emerging signs of resistance against CQ were observed [6, 16, 17]. Following the Abuja Summit of 2000, Guinea developed its first strategic plan (2001–2005) to achieve the Abuja targets of reducing morbidity and mortality in children less than 5 years by 50%—an objective that eventually remained unfulfilled. After organizational restructuring, the national malarial control programme (NMCP) was formally created in 2003 with the mission to implement policies to combat malaria burden.

**Table 1:** Major milestones in malaria control activities in Guinea

Years	Milestones
1958	Guinea gained independence
1970	Development of a policy document to fight against malaria Chloroquine (CQ) introduced as a front-line drug Implementation of spraying of insecticides as a vector control measure
1975	Entomological and parasitological studies conducted in Bentourayah and Béréiré in Coyah and Forécariah with WHO’s support
1987	Integrated project to fight against communicable childhood diseases (CCCD) including malaria introduced in the health districts of Conakry, Kindia and Télimélé CQ adopted as a prophylaxis in pregnant women
2001	Development of national policy against malaria on the recommendations of the Abuja Summit in 2000 The first strategic plan (2001–2005) drafted with the aim to reduce morbidity and mortality in children under 5 by 50%
2003	Creation of the national programme to fight against malaria
2004–2005	A randomised trial assessing the efficacy of ACTs conducted in Dabola [18]
2005	Adoption of ACT as the first-line treatment of uncomplicated falciparum malaria SP adopted as intermittent preventive treatment (IPT) in pregnant women The second strategic plan (2006–2010) drafted with aim of scaling up ACTs, RDTs, and LLINs
2008	Revision and adoption of the national policy document for the fight against malaria
2009	Implementation of the first mass distribution campaign for LLINs
2011	Guinea is part of the US President’s malaria initiative (US PMI)
2012–2016	The third strategic plan (2013–2017) drafted with the aim of scaling up to the community level diagnosis using the RDTs, the management of uncomplicated malaria by ACT, and severe cases using artemisinin derivatives Two national campaigns for the mass distribution of LLINs carried out The strengthening of IPT and the implementation of two chemoprevention campaigns

Source: National malaria control programme of Guinea [61]

*ACT* artemisinin-based combination therapy, *RDTs* Rapid diagnostic tests, *LLINs* long-lasting insecticidal nets, *SP* sulfadoxine-pyrimethamine, *CQ* chloroquine

During the 2000s, CQ resistance was rampant and widespread (as described by Bonnet et al. [18]). In the wake of the relentless CQ resistance, two studies in Central Guinea tested the field efficacy of artemisinin-based combinations under the auspices of Médecins Sans Frontières [18]. The studies evaluated efficacy of artesunate + amodiaquine (AS + AQ) and artesunate + SP (AS + SP); both these regimens were found to be highly efficacious. These results led to Guinea formally adopting artemisinin-based combination therapy (ACT) in 2005 as the first-line treatment for uncomplicated falciparum malaria. In addition, SP replaced CQ for intermittent preventive treatment (IPT) in pregnant women.

A second strategic plan (2006–2010) was adopted following the 2006 Abuja declaration with a target to achieve universal access to basic care. This led to scaling-up of nationwide distribution of ACT, rapid diagnostic tests (RDTs) and LLINs through the support of Global Fund. During 2010–2019 period, “test before treat” programme was adopted and scaled up, two national campaigns of mass distribution of LLINs were carried out (in 2013 and 2016), and two seasonal malaria chemoprevention programme were also implemented. Over 27.6 million pyrethroid-treated LLINs were distributed (2013–2017) [19]. In 2011, Guinea was added to the United States’ President’s Malaria Initiative (US PMI) list of high malaria burden countries—the US PMI has provided critical technical and financial support in combatting the high burden of malaria.

### Therapeutic efficacy studies against uncomplicated P. falciparum malaria: the past and the present

Relevant studies describing anti-malarial drug efficacy in Guinea were identified by searching the publications indexed in the WorldWide Antimalarial Resistance Network (WWARN) library of clinical studies [20]. The WWARN library is a periodically updated living systematic review that indexes all published anti-malarial trials from 1946 onwards. Additional studies were identified by searching the references of the included studies and by conducting further search on PubMed to identify more recent studies using a broad search terms of (malaria) AND (Guinea). Information on early and late parasitological responses were extracted from eligible studies (Table 2). Clinical efficacy of anti-malarial drugs used in the past (Chloroquine era: 1970–2005) and the present (ACT era: 2005–date) is summarized next.

**Table 2:** In vivo efficacy studies in Guinea

Drug	Posology	Study period	Location	Age-range	Number of patients	Parasite positivity rates (PPRs)	Efficacy outcome
**Chloroquine**							
Turaman-1992 [6]	10 mg/kg on day0 and day1 and 5 mg/kg on day2	1991	Kouroussa	< 14 years	44	Day1 PPR: 100% Day2 PPR: 100% Day7 PPR: 27% (12/44)	32 (73%) showed a good response (RI); 12 (27%) with RI/RII resistance on day 7
WHO-2005 [25] (the report summarizes data from 8 in vivo studies)	–	1996–2001	–	–	–	–	Median failure rate of 15.6% [range: 7.7–28.3%]
Loua-2017 [26]	10 mg/kg on day0 and day1 and 5 mg/kg on day2	2007	Dubreka	5–15 years	24	^a^	Only 4/21 (19%) patients achieved parasite clearance by day28
**Artesunate-amodiaquine**							
Bonnet-2007 [18]	AQ dose: 30 mg/kg over 3 days AS dose: 12 mg/kg AS over 3 days	2004	Dabola	6–59 months	110	–	Day28 PCR corrected efficacy: 99.5% [95% CI: 94.7–99.8]
WANECAM-2018 [28]	25:67.5 mg tablet: (≥ 5 to < 9 kg 1 tablet); 50:135 mg tablet:(≥ 9 to < 18 kg 1 tablet); 100:270 mg tablet: (> 18 to < 36 kg 1 tablet; ≥ 36 kg 2 tablets)	2011–2016	Mafèrinyah	≥ 6 months	311	–	Day28 efficacy: 100%^b^ Day42 efficacy: > 99%^b^
Beavogui-2020 [5]	–	2016	Mafèrinyah	6–59 months	107	Day2 PPR: 10.5% Day3 PPR: 1%	100% (day28 K-M estimates)
Beavogui-2020 [5]	–	2016	Labé	6–59 months	104	Day2 PPR: 4.8% day3 PPR: 0%	day28 efficacy: 99% [95% CI: 97.2–100] (K-M estimates)
**Artesunate-SP**							
Bonnet-2007 [18]	–	2004	Dabola	6–59 months	110	–	Day28 efficacy: 99.0% [95% CI: 94.5–99.8] (PCR corrected)
**Artemether-lumefantrine**							
Beavogui-2020 [5]	–	2016	Mafèrinyah	6–59 months	104	Day2 PPR: 11.9% Day3 PPR: 0%	Day28 efficacy: 100% (K–M estimates)
Beavogui-2020 [5]	–	2016	Labé	6–59 months	105	Day2 PPR: 9.7% Day3 PPR: 0%	Day28 efficacy: 99.0% [95% CI: 97.1–100] (K–M estimates)
**Pyronaridine Artesunate**							
WANECAM-2018 [28]	60:20 mg sachet: (5 to < 8 kg 1 sachet; 8 to < 15 kg 2 sachets; 15 to < 20 kg 3 sachets); 180:60 mg sachet: (20 to < 24 kg 1 sachet; 24 to < 45 kg 2 sachets; 45 to < 65 kg 3 sachets; ≥ 65 kg 4 sachets)	2011–2016	Mafèrinyah	≥ 6 months	235	–	Day28 efficacy: 100%^b^ Day42 efficacy: 99%^b^
**Dihydroartemisinin piperaquine**							
WANECAM-2018 [28]	20:160 mg tablet (5 to < 7 kg 1/2 tablet; 7 to < 13 kg 1 tablet); 40:320 mg tablet (13 to < 24 kg 1 tablet; 24 to < 36 kg 2 tablets; 36 to < 75 kg 3 tablets)	2011–2016	Mafèrinyah	≥ 6 months	309	–	Day28 efficacy: 99.8%^b^ Day42 efficacy: 99.5%^b^

*CQ* chloroquine, *AQ* amodiaquine, *AS* artesunate, *K–M* Kaplan–Meier estimates, *PCR* polymerase chain reaction used for distinguishing new infection from recrudescence, *CI* Confidence interval

^a^Only 4 (19%) of the 21 patients had their parasite clearance cleared on day 28

^b^Repeated episodes are included. All treatments met WHO efficacy criteria (> 95% ACPR) for therapy adoption, with PCR-adjusted ACPR in the per protocol population at least 99.5% at day28 and at least 98.6% at day42

#### Chloroquine

CQ was the mainstay treatment against uncomplicated malaria in the 1960s. Despite reports of decreased CQ susceptibility in the neighbouring countries during the 1960s, *P. falciparum* infections were found to respond well to CQ therapy in Guinea [21, 22] and the regimen was adopted as the frontline drug in the first malaria policy document in 1970 (Table 1). By the late 1980s, there was a sharp decline in parasite susceptibility to CQ (in parallel, CQ resistance was confirmed in some West African countries) [23]. Several clinical studies conducted in Kindia and in Conakry in the 1990s found no evidence of CQ resistance when patients were treated with 25 mg/kg CQ dose over 3 days [24]. Although in vitro studies had identified resistant isolates of *P. falciparum* during late 1980s and early 1990s, there was no in vivo evidence of CQ resistance [6, 16, 17]. Evidence of partial CQ resistance emerged in 1991 in a study conducted among 44 children at Kouroussa district hospital [6]. During 1996–2001, 8 in vivo studies were conducted to assess the efficacy of CQ and a median failure rate of 15.6% [range: 7.7–28.3] was observed [25]. By the turn of the millennium, failure rates against CQ were greater than 20% (described by Bonnet et al*.* in 2006 [18]). Lack of feasible alternatives meant that CQ continued to be used as the first-line drug against *P. falciparum* into the early 2000s. A study in 2007 reported that only 4/21 (19%) patients achieved parasite clearance by day 28 after 25 mg/kg CQ dose [26]. The drug is no longer recommended after the adoption of ACT as first-line therapy.

#### Artemisinin-based combination therapy

The artesunate + SP (AS + SP) regimen was one of the early artemisinin-based combinations used in Guinea. It was used in the Lainé refugee camp in the forest region for over two years in the early 2000s [18]. The regimen was also tested in a clinical study of 110 patients in Dabola (2004) with a day 28 polymerase chain reaction (PCR) corrected efficacy of 99.0% [95% confidence interval (CI): 94.5–99.8] (Table 2).

ACT was formally adopted as the first-line treatment for uncomplicated malaria by the NMCP in 2005, with artesunate + amodiaquine (AS + AQ) as the preferred regimen [27]. The efficacy of AS + AQ was initially tested in Dabola (2004) and has been further evaluated in Mafèrinyah (2011–2016) and Labé (2016) in a total of 632 patients (Table 2) [18, 28, 29]. Rapid parasitological responses has been observed following this regimen with day 3 parasite positivity rate (PPR) of 1% in Mafèrinyah and 0% in Labé in 2016 (PPRs were not reported in the Dabola study).

Artemether-lumefantrine (AL) has now largely replaced AS + AQ as the preferred regimen since 2016 [29]. The efficacy of the AL regimen has been evaluated in a single study in 2016 (209 patients) at two sites (Mafèrinyah and Labé) [5]. The PCR corrected efficacy on day 28 at both sites were ≥ 99% and parasite positivity rates were approximately 10% on day 2 and 0% on day 3 (Table 2).

Other artemisinin-based combinations tested include pyronaridine-artesunate (PA) (n = 235 patients) and dihydroartemisinin-piperaquine (DP) (n = 309 patients) evaluated in Mafèrinyah (2011–2016) with a day 28 PCR corrected efficacy > 95% for both regimens (Table 2).

#### Other anti-malarial drugs

Quinine is now mainly used as a rescue therapy as outlined in the NMCP protocol [30]. Low sensitivity against quinine was reported in 10 isolates tested in 1986 [16] and in a traveller in 2004 [31]. Current therapeutic efficacy or drug resistance status on quinine remains unknown.

Sulfadoxine-pyrimethamine remains the current first-line therapy for treatment of malaria in pregnancy [3] and is also adopted as intermittent preventive treatment (IPT) among pregnant women [30]. Further data on in vivo efficacy of SP is not available. However, there were no known reports of resistance against SP in Guinea until 2004 (See Table 1.3 in thesis by Amin A.A. [32]). Mutations in dihydrofolate reductase (*dhfr*) and dyhydropteroate synthetase (*dhps*) genes in isolates tested after 2004 is presented in Table 3.

**Table 3:** Studies describing prevalence of molecular markers of antimalarial resistance

Markers	Year	Study site	Marker type	Total tested	Number of mutations	Mutation percentage [95% CI]
***dhps***						
Bonnet-2007 [18]	2004	Lainé refugee camp	Wild-type	114	8	7.0% [3.6–13.2%]
Bonnet-2007 [18]	2004	Lainé refugee camp	Single mutant 436	114	31	27.2% [19.9–36%]
Bonnet-2007 [18]	2004	Lainé refugee camp	Single mutant 437	114	38	33.3% [25.3–42.4%]
Bonnet-2007 [18]	2004	Lainé refugee camp	Double mutant 436–437	114	29	25.4% [18.3–34.1%]
Bonnet-2007 [18]	2004	Lainé refugee camp	Double mutant 437–540	114	8	7.0% [3.6–13.2%]
Xu-2019 [36]	2013–2016	Travellers	I431V	13	0	0.0% [0.0–22.8%]
Xu-2019 [36]	2013–2016	Travellers	S436A/F	13	1	7.7% [1.4–33.3%]
Xu-2019 [36]	2013–2016	Travellers	A437G	13	13	100% [77.2–100%]
Xu-2019 [36]	2013–2016	Travellers	K540E	13	2	15.4% [4.3–42.2%]
Xu-2019 [36]	2013–2016	Travellers	A581G	13	0	0.0% [0.0–22.8%]
Xu-2019 [36]	2013–2016	Travellers	A613S	13	1	7.7% [1.4–33.3%]
***dhf***						
Bonnet-2007 [18]	2004	Lainé refugee camp	Wild-type	148	20	13.5% [8.9–20.0%]
Bonnet-2007 [18]	2004	Lainé refugee camp	Double mutant 59–108	148	2	1.4% [0.4–4.8%]
Bonnet-2007 [18]	2004	Lainé refugee camp	Triple mutant 51–59–108	148	126	85.1% [78.5–90.0%]
Xu-2019 [36]	2013–2016	Travellers	N51I	13	12	92.3% [66.7–98.6%]
Xu-2019 [36]	2013–2016	Travellers	C59R	13	12	92.3% [66.7–98.6%]
Xu-2019 [36]	2013–2016	Travellers	S108N	13	13	100% [77.2–100%]
Xu-2019 [36]	2013–2016	Travellers	51I-59R-108 N	13	12	92.3% [66.7–98.6%]
***dhps* and** ***dhfr*** **combined**						
Bonnet-2007 [18]	2004	Lainé refugee camp	Quintuple mutant: *dhfr* 51–59–108 and *dhps* 437–540	110	8	7.3% [3.7–13.7%]
Bonnet-2007 [18]	2004	Lainé refugee camp	Quintuple mutant: dhfr 51–59–108 and *dhps* 436–437	110	27	24.5% [17.5–33.4%]
Bonnet-2007 [18]	2004	Lainé refugee camp	*dhfr* 51–59–108 and *dhps* single mutant	110	54	49.1% [39.9–58.3%]
Bonnet-2007 [18]	2004	Lainé refugee camp	Three or less mutations	110	21	19.1% [12.8–27.4%]
Xu-2019 [36]	2013–2016	Travellers	51I + 59R + 108N + 437G (IRNG)	13	8	61.5% [35.5–82.3%]
Xu-2019 [36]	2013–2016	Travellers	51I + 59R + 108N + 437G + 540E (IRNGE)	13	2	15.4% [4.3–42.2%]
Xu-2019 [36]	2013–2016	Travellers	51I + 59R + 108N + 437G + 540E + 581G or 613S (IRNGEG/S)	13	0	0.0% [0.0–22.8%]
***pfcrt***						
Durand-2001 [38]	1995–1999	Travellers	*pf*crt 76T	1	0	0.0% [0.0–79.3%]
Durand-2001 [38]	1995–1999	Travellers	*pf*crt K76	1	1	100.0% [20.7–100%]
Andriantsoanirina-2010 [37]	2003	Travellers	*pf*crt 76T	1	1	100.0% [20.7–100%]
Andriantsoanirina-2010 [37]	2003	Travellers	*pf*crt K76	1	0	0.0% [0.0–79.3%]
Zhou-2016 [39]	2012–2015	Conakry	*pf*crt CMNK	33	21	63.6% [46.6–77.8%]
Zhou-2016 [39]	2012–2015	Conakry	*pf*crt CIET	33	11	33.3% [19.8–50.4%]
Zhou-2016 [39]	2012–2015	Conakry	*pf*crt K76	33	21	63.6% [46.6–77.8%]
Zhou-2016 [39]	2012–2015	Conakry	*pf*crt 76T	33	11	33.3% [19.8–50.4%]
Lu-2017 [40]	2011–2014	–	*pf*crt K76	7	3	42.9% [15.8–75.0%]
Lu-2017 [40]	2011–2014	–	*pf*crt 76T	7	4	57.1% [25.0–84.2%]
Lu-2017 [40]	2011–2014	–	*pf*crt 76K/T	7	0	0.0% [0.0–35.4%]
Lu-2017 [40]	2011–2014	–	*pf*crt CMNK	7	3	42.9% [15.8–75.0%]
Lu-2017 [40]	2011–2014	–	*pf*crt CIET	7	4	57.1% [25.0–84.2%]
Tao-2018 [41]	2012–2016	Conakry	*pf*crt 76T	10	3	30.0% [10.8–60.3%]
Tao-2018 [41]	2012–2016	Conakry	*pf*crt K76	10	7	70.0% [39.7–89.2%]
***pfmdr1***						
Witkowski-2010 [43]	2005–2009	Travellers	Copy number > 1	9	0	0.0% [0.0–29.9%]
Durand-2001 [38]	1995–1999	Travellers	*pf*mdr1 86Y	1	1	100.0% [20.7–100%]
Durand-2001 [38]	1995–1999	Travellers	*pf*mdr1 N86	1	0	0.0% [0.0–79.3%]
Tao-2018 [41]	2012–2016	Conakry	*pf*mdr1 86Y	10	2	20.0% [5.7–51.0%]
Yang-2019 [42]	2012–2016	Conakry	*pf*mdr1 NFSND	33	14	42.4% [27.2–59.2%]
Yang-2019 [42]	2012–2016	Conakry	*pf*mdr1 YYSNY	33	0	0.0% [0.0–10.4%]
Yang-2019 [42]	2012–2016	Conakry	*pf*mdr1 N86	33	14	42.4% [27.2–59.2%]
Yang-2019 [42]	2012–2016	Conakry	*pf*mdr1 86Y	33	19	57.6% [40.8–72.8%]
Beavogui-2020 [5]	2016	Mafèrinyah and Labé	*pf*mdr1 86N	379	296	78.1% [73.7–82%]
Beavogui-2020 [5]	2016	Mafèrinyah and Labé	*pf*mdr1 86Y	379	59	15.6% [12.3–19.6%]
Beavogui-2020 [5]	2016	Mafèrinyah and Labé	*pf*mdr1 86N/Y	379	24	6.3% [4.3–9.2%]
Beavogui-2020 [5]	2016	Mafèrinyah and Labé	*pf*mdr1 184Y	379	117	30.9% [26.4–35.7%]
Beavogui-2020 [5]	2016	Mafèrinyah and Labé	*pf*mdr1 184 F	379	206	54.4% [49.3–59.3%]
Beavogui-2020 [5]	2016	Mafèrinyah and Labé	*pf*mdr1 184 Y/F	379	56	14.8% [11.6–18.7%]
Beavogui-2020 [5]	2016	Mafèrinyah and Labé	*pf*mdr1 1246 D	370	363	98.1% [96.1–99.1%]
Beavogui-2020 [5]	2016	Mafèrinyah and Labé	*pf*mdr1 1246 Y	370	6	1.6% [0.7–3.5%]
Beavogui-2020 [5]	2016	Mafèrinyah and Labé	*pf*mdr1 1246 D/Y	370	1	0.3% [0.0–1.5%]
**pfk13**						
Beavogui-2020 [5]	2016	Mafèrinyah and Labé	Wild-type	389	380	97.7% [95.7–98.8%]
Beavogui-2020 [5]	2016	Mafèrinyah and Labé	Mutant	389	9	2.3% [1.2–4.3%]

**Source:** The table is derived from the open-access library of molecular markers of antimalarial resistance indexed in the WorldWide Antimalarial Resistance Network systematic review libraries [34, 35]

*CI* confidence interval; 95% CI was derived using Wilson’s method

### Therapeutic efficacy studies against severe P. falciparum malaria

The estimated incidence of in-patient severe malaria in Guinea is 115.6 cases per 100,000 person per year [33]. No published studies describing the efficacy of anti-malarial drugs against severe malaria in Guinea were identified. Parenteral quinine was the first-line regimen for the treatment of severe malaria prior to its replacement by artesunate therapy [3, 30]. A case of severe malaria was described in a traveller who was successfully treated with quinine [8].

### Therapeutic efficacy studies against non-falciparum malaria

There is no specific policy for the treatment of vivax or other non-falciparum malaria [3]. Reports of non-falciparum malaria are rare and are described mostly among travellers. CQ and primaquine therapy was used for treating a case of traveller vivax malaria with good clinical outcome [9].

### Studies describing molecular markers of drug resistance

The WorldWide Antimalarial Resistance Network (WWARN) database of molecular markers of resistance against partner drugs for ACT and for SP were searched for identifying publications from Guinea [34, 35]. A recently published study was identified from a further literature search carried out by using the search terms of (malaria) AND (Guinea) on PubMed [5]. Reported mutations are summarized in Table 3 and some key results are presented below.

#### Dihydrofolate reductase (*dhfr*) and dihydropteroate synthetase (*dhps*)

In 2004, single or double mutations in *dhps* 436–437 or *dhps* 437–540 were found in 93% (106/114) and *dhfr* 59–108 or *dhfr* 51–59–108 mutations in 86.5% (128/148) of the isolates tested in the Lainé refugee camp [18]. Molecular data from 13 travellers (2013–2016) indicated 92.3% (12/13) harboured triple *dhfr* (51–59–108) mutations [36]. All the isolates also harboured single mutation in *dhps* 437 gene with *dhps* 436 present in 7.7% (1/13) and *dhps* 540 in 15.4% (2/13).

#### Plasmodium falciparum chloroquine resistance transporter (pfcrt)

*pfcrt* K76 was identified in travellers in the late 1990s and early 2000s [37, 38]. During 2012–2015, a study reported the presence of *pfcrt* 76T allele in 33.3% (11/33) travellers [39]. Data from travellers continue to indicate the presence of mutations in the *pfcrt* genes [39–42].

#### *Plasmodium falciparum* multi-drug resistance 1 (*pfmdr1*)

*pfmdr1* N86 mutations were reported in 78.1% (298/379) of the isolates tested in a clinical study carried out in Mafèrinyah and Labé in 2016 [5] (Table 3). Among travellers, a study reporting data from 2005–2009 found no copy number elevations in *pfmdr1* gene [43]. Another study reported *pfmdr1* N86 mutations in 42.4% (14/33) of the travellers (2012–2016) [42].

#### *Plasmodium falciparum* kelch-13 (*pfk13*)

In 2016, isolates from patients in Mafèrinyah and Labé found that 2.3% (9/389) harboured *kelch-13* mutations [5]. The reported mutations however are currently not known to be associated with resistance against artemisinin.

## Discussion

Despite ACT being the first-line therapy for more than 15 years, only three published in vivo trials were identified (1495 patients, conducted from 2004 to 2016). Data from these three trials demonstrate rapid clearance of parasites and an efficacy greater than 95%—as expected for ACT [44–48]. In particular, the three studies described data collected up to 2016 and there is a paucity of in vivo parasite susceptibility from 2017 onwards. The NMCP has begun therapeutic efficacy studies in four sentinel sites to monitor drug efficacy and track early signs of drug resistance [29].

In contrast to the paucity of data on drug efficacy, studies describing molecular markers were relatively more common (Table 3). Mutations were reported in *pfmdr1* N86 in 78.1% (298/379) from Mafèrinyah and Labé in 2016 [5]. Mutation in *pfmdr1* allele is associated with increased failure for ACT with lumefantrine as partner drug [49]. Monitoring mutations in *pfmdr1* gene including assessment of copy number elevation is, therefore, critical for the Guinean malaria control since AL has now largely replaced AS + AQ as a front line drug [29]. One study described mutations in the *kelch-13* gene [5] and reassuringly, all the mutations identified are currently known not be associated with artemisinin resistance.

In the past 20 years, Guinea has made important progress towards combatting malaria, including implementation of several mass drug administration campaigns, distribution of LLINs, improved access of RDTs and ACT. All these achievements are yet to materialize in a visible reduction in case burden; approximately 3 million cases still occur annually. A major challenge is that despite improved treatment access and coverage, the adoption of ACT could be actually sub-optimal. Among children less than 5 years of age with fever and malaria, the estimated uptake of ACT was only 1.2% [95% CI: 0.6–2.1%] in 2015 [50]. Awareness campaigns and public/community engagement activities might, therefore, be needed [51]. An integrated approach that focuses on overall aspect of malaria epidemiology, including vector control measures such as promotion of environmental sanitation to reduce the breeding sites for mosquitoes in addition to the periodic assessment of drug efficacy, and continuous surveillance of molecular markers across wide geographic span is likely to be key to reducing the overall case burden.

There are also several immediate challenges. First, the ongoing COVID-19 pandemic remains a major immediate threat. Unlike the 2014–2015 Ebola Virus Disease (EVD) outbreak that devastated control activities only in some regions, the ongoing COVID-19 pandemic has disrupted control activities throughout the country. The number of suspected malaria cases has increased by 2.6% (from 3,334,355 in 2019 to 3,422,309 in 2020) and the number of rapid diagnostic test (RDTs) confirmed positive cases has increased by 10.9% (from 1,801,694 in 2019 to 1,998,329 in 2020) (Eugene Kaman Lama, NMCP, pers. commun.). The full impact of the pandemic on malaria control activities cannot be clearly assessed at this moment, as the future course of the pandemic remains unknown. For example, during the 2014–2015 EVD outbreak, there was an estimated 45% increase in untreated malaria cases and 5,600 [95% CI: 3000–11,100] additional malaria attributed deaths [52]. Although the current data has shown only moderate increase in malaria cases (in 2020 compared to 2019), the impact is likely to be substantial if the ongoing COVID-19 pandemic leads to persistent interruption of the control activities for a longer period. Second, counterfeit/sub-standard/falsified medicines is a major public health problem and has been under-recognized. Reports of fake chloroquine and halofantrine in circulation were documented in 2009 [53]. Despite several crackdowns from the government, counterfeit medicines can easily be purchased in the Niger and Madina markets in Conakry [54, 55]. The government seized more than 300 containers of fake medicines in Conakry port in 2019—this likely indicates the scale of widespread availability of fake drugs [56]. It is reported that more than 100,000 Guineans die annually due to fake or falsified drugs [57]. Guinea is a recent signatory on a treaty for the establishment of African Medicines Agency (AMA). The AMA has an overarching objective to improve access to quality, safe and efficacious medical products throughout the continent [58]. Such collaborative initiative is important for the control of sub-standard medicines in the entire region.

Finally, the recent report of the identification of a de novo emergence of Kelch-13 mediated artemisinin-resistance in Rwanda is a major concern [59, 60], but a recent study conducted in Mafèrinyah and Labé has not identified any mutations that are currently known to be associated with artemisinin resistance [5]. Continuous monitoring of the therapeutic efficacy of the existing front line drugs and conducting molecular surveillance studies to generate complementary information on drug resistance remains crucial.

## Conclusion

There is limited data on in vivo efficacy of ACT regimens in Guinea despite their adoption as first-line treatment for 15 years. Annual case burden still remains very high with the entire population at risk of malaria. The threat of COVID-19 pandemic and the widespread availability of counterfeit medicines remains major immediate challenges.

## Data Availability

All the data used are available within tables and figures presented in the manuscript.

## Abbreviations

ACT: Artemisinin-based combination therapy
AL: Artemether-lumefantrine
AS + AQ: Artesunate-amodiaquine
AS + MQ: Artesunate-mefloquine
CI: Confidence interval
CQ: Chloroquine
DP: Dihydroartemisinin-piperaquine
IRS: Indoor residual spray
ITNs: Insecticide-treated nets
LLINs: Long-lasting insecticidal nets
NMCP: National malaria control programme
PCR: Polymerase chain reaction
RDTs: Rapid diagnostic tests
SP: Sulfadoxine-pyrimethamine
TES: Therapeutic efficacy study
WWARN: WorldWide Antimalarial Resistance Network
